# Enterotypes of the Gut Microbial Community and Their Response to Plant Secondary Compounds in Plateau Pikas

**DOI:** 10.3390/microorganisms8091311

**Published:** 2020-08-28

**Authors:** Chao Fan, Liangzhi Zhang, Haibo Fu, Chuanfa Liu, Wenjing Li, Qi Cheng, He Zhang, Shangang Jia, Yanming Zhang

**Affiliations:** 1 Key Laboratory of Adaptation and Evolution of Plateau Biota, Northwest Institute of Plateau Biology, Chinese Academy of Sciences, Xining 810008, China; fanchao@nwipb.cas.cn (C.F.); lzzhang@nwipb.cas.cn (L.Z.); fu_haibo@163.com (H.F.); Liuchuanfa@yandex.com (C.L.); wjli@nwipb.cas.cn (W.L.); chengqi@nwipb.cas.cn (Q.C.); ZhangHenwipb@yandex.com (H.Z.); 2 Qinghai Provincial Key Laboratory of Animal Ecological Genomics, Xining 810008, China; 3 University of Chinese Academy of Sciences, Beijing 100049, China; 4 College of Grassland Science and Technology, China Agricultural University, Beijing 100193, China

**Keywords:** gut microbiota, 16S rDNA, enterotype, plant secondary compound, adaptability, microbial diversity, plateau pika

## Abstract

Animal gut microbiomes can be clustered into “enterotypes” characterized by an abundance of signature genera. The characteristic determinants, stability, and resilience of these community clusters remain poorly understood. We used plateau pika (*Ochotona curzoniae*) as a model and identified three enterotypes by 16S rDNA sequencing. Among the top 15 genera, 13 showed significantly different levels of abundance between the enterotypes combined with different microbial functions and distinct fecal short-chain fatty acids. We monitored changes in the microbial community associated with the transfer of plateau pikas from field to laboratory and observed that feeding them a single diet reduced microbial diversity, resulting in a single enterotype with an altered composition of the dominant bacteria. However, microbial diversity, an abundance of some changed dominant genera, and enterotypes were partially restored after adding swainsonine (a plant secondary compound found in the natural diet of plateau pikas) to the feed. These results provide strong evidence that gut microbial diversity and enterotypes are directly related to specific diet, thereby indicating that the formation of different enterotypes can help animals adapt to complex food conditions. Additionally, natural plant secondary compounds can maintain dominant bacteria and inter-individual differences of gut microbiota and promote the resilience of enterotypes in small herbivorous mammals.

## 1. Introduction

Mammalian gut microbiota display different community structures depending on various factors, such as diet, age, and geography [1,2]. The composition of gut microbiota can vary among individuals based on their habitat and seasons of the year [3,4]. Studies on population stratification have suggested that the human gut microbiota is classified under the following three distinct categories or “enterotypes”: *Bacteroides*, *Prevotella*, and *Ruminococcus* [5,6]. Analysis of the dominant genera has revealed significant functional variations associated with microbial composition. For example, the *Bacteroides*-dominant enterotype is strongly associated with a diet rich in animal proteins and saturated fats [7], whereas the *Prevotella*-dominant enterotype is typical of a fiber-rich diet [8,9]. Subsequently, enterotypes have been found in several species, such as mice (*Mus musculus*) [10], pigs (*Susscrofa domestica*) [11], and bumblebees (*Bombus* spp.) [12], although their gut composition is distinct from that of human beings [13]. However, there is a lack of knowledge on the determinants of gut community composition and enterotypes in plateau herbivorous animal populations [14].

A few studies have reported that environmental interventions such as geographical conditions, antibiotics, and plant secondary compounds (PSCs) may cause considerable alteration in the microbial ecosystem of the mammalian gut [15,16,17]. Meanwhile, enterotypes appear to have a tendency of recovering and returning to the original state after external disturbance [14,15,18]. A study reported that wild-type mice quickly change their enterotype classification upon transfer to a laboratory with a single diet [19]. It is worth noting that short-term antibiotic treatment can cause partially recoverable shifts in the gut microbiota of humans [20,21]. In immigrants, the dominant species of gut microbiota usually change based on variations in the environment and diet [22]. Liu et al. [23] reported that, even after staying in Africa for half a year, the enterotypes of Chinese doctors reverted to normal after the subjects reverted to their routine diets. However, there is limited data on changes in gut microbial composition in animals that make it difficult to draw conclusions regarding enterotype resilience [14].

Plants produce a wide array of secondary compounds that are metabolic by-products [24]. These compounds play a pivotal role in the plant’s defense mechanism via agonistic or antagonistic interactions with beneficial microbes or pathogens [25]. Therefore, these compounds are one of the most important factors affecting the adaptive evolution of herbivores [24,26]. Freeland and Janzen have hypothesized that exposure to PSCs modifies herbivore-associated microbial community composition [27]. Studies on insects and small herbivorous mammals have found that PSCs not only present a potential lethal risk and exert an antibacterial effect on the host but also significantly alter the gut microbial community structure and promote microbial diversity and retention of the native gut microbiota in the host [26,28,29]. As these studies have not been considered in the enterotype framework, there remains a lack of understanding of whether PSCs can cause inter-individual differences and determine the enterotypes of the gut microbiota.

Plateau pikas (*Ochotona curzoniae*) are diurnal, burrowing, non-hibernating, small herbivorous mammals distributed mostly within the high alpine grasslands of the Qinghai–Tibet Plateau [30]. Significant differences in their gut microbiota have been documented between geographical populations, thereby indicating strong disparities in population density and physiological characteristics [31,32]. Plants of the *Oxytropis* genus represent an important diet item for wild plateau pikas and account for a large proportion of their natural feed [33,34]. These plants are called “locoweed” as they contain swainsonine (SW), which is neurotoxic to livestock [35].

In this study, using plateau pikas as the animal model, we explored whether herbivorous mammals living in the alpine meadow harbored enterotype-like structures and whether SW, a typical PSC, played a role in maintaining dominant bacterial genus, thus preserving native microbial communities and enhancing the diversity of gut microbes. We also determined whether SW caused inter-individual differences of the gut microbiota and promoted the restoration of enterotypes. This study represents a step forward in understanding the interactions between PSCs and gut microbiota in small herbivorous mammals.

## 2. Materials and Methods

### 2.1. Sample Collection

All animal procedures were conducted according to the Regulations for the Administration of Laboratory Animals established by the Ministry of Science and Technology of the People’s Republic of China (2017 Revision). Wild plateau pika sample collection was performed in spring (April), summer (July), autumn (November), and winter (January) between July 2015 and April 2018 in Gangcha County (Altitude: 3650 m; N: 37°9′39″, E: 100°28′40″). The area is covered by typical alpine meadow and is characterized by a plateau continental climate with an average annual temperature of 2 °C [36]. We live-trapped adult plateau pikas and locked them in cages previously sterilized with 75% alcohol. Fresh feces were collected in 2-mL tubes (Sigma-Aldrich, St. Louis, MO, USA), immediately frozen, and stored in liquid nitrogen before being sent for analysis to the Northwest Plateau Institute of Biology, Chinese Academy of Sciences, Xining, Qinghai. A total of 126 wild plateau pikas were sampled.

### 2.2. Determination of Fecal SCFAs

Four short-chain fatty acids (SCFAs) (acetic acid, propionic acid, i-butyric acid, and n-butyric acid) of 55 fecal samples were measured using propyl chloroformate (PCF) derivatization followed by gas chromatography-mass spectrometry (GC-MS) [37]. Each fecal sample (~100 mg) was added to 1000 μL of 0.005 M aqueous NaOH containing IS (Internal standard, 5 µg/mL caproic acid -d3), and the suspension was homogenized for 10 min and centrifuged at 13,000 rpm at 4 °C for 20 min. A 500 μL aliquot of the supernatant was transferred into a 15 mL BBI^®^ topped cap centrifuge tube (F600888, BBI Life Sciences Corporation, Shanghai, China). Then, 300 μL of water, 500 μL of PrOH/Py solution (3:2, v/v), and 100 μL of PCF were added to this aliquot and subsequently vortexed for 30 min. The derivatization reaction proceeded under ultrasonication for 1 min. With 300 µL hexane added in the first extraction, the reaction mixture was vortexed for 1 min, followed by centrifugation at 2000 rpm for 5 min, and 300 μL of the supernatant of the hexane layer was transferred to an autosampler vial. After the second extraction with another 200 µL of hexane, a total of 500 μL of the derivatized extract was collected in the autosampler vial. Approximately 10 mg of anhydrous sodium sulfate was added to remove traces of water from the hexane in the autosampler vial. The mixture was briefly vortexed prior to GC-MS analysis. An Agilent 7890A/5975C GC-MS (MSD, Agilent Technologies, Santa Clara, CA) was used to perform the analysis. Derivatives were separated using an HP-5MS capillary column (Agilent J&W Scientific, Folsom, CA), 1 mL of the derivatized sample was injected in split mode (split ratio: 10:1), and the solvent delay time was set to 2.5 min. The initial oven temperature was maintained at 50 °C for 2 min, increased to 70 °C at a rate of 10 °C min^−1^, to 85 °C at 3 °C min^−1^, to 110 °C at 5 °C min^−1^, and to 290 °C at 30 °C min^−1^, and finally held at 290 °C for 8 min. The carrier gas was helium, and the constant flow rate was 1 mL min^−1^. The front inlet, transfer line, and electron impact (EI) ion source temperatures were set at 260 °C, 290 °C, and 230 °C, respectively. The electron energy was −70 eV, and full scan mode (m/z 30–600) was used to collect the mass spectral data.

### 2.3. Animal Maintenance and Diet Treatment

After collecting wild fecal samples, 10 animals captured in July 2017 and 10 captured in November 2017 were brought to the laboratory. Each plateau pika was kept in an opaque plastic box (45 cm × 32 cm × 19 cm) alone, with natural light and free access to purified water and 45 g of pellet feed (Rabbit maintenance feed, KEAO XIELI Feed Co., Ltd. Beijing) daily. The main components of feed are mentioned in Table S1. Ten plateau pikas captured in July 2017 (Group J) were reared in the laboratory for 20 weeks (long-term intervention) and the second fecal sample was taken thereafter. Ten plateau pikas captured in November 2017 (Group N) were reared in the laboratory for 2 weeks (short-term intervention) and the second fecal sample was collected thereafter. At this point, SW was added to the daily diet and the animals were reared on it for 4 weeks prior to the third fecal sample collection. Wild fecal samples of Group J were defined as JW. The fecal samples from the second and third collections were designated as JC (fecal samples of captive individuals in Group J) and JSW (fecal samples of captive individuals fed with SW in Group J), respectively. Similarly, samples of Group N were divided into NW (wild fecal samples of Group N), NC (fecal samples of captive individuals in Group N), and NSW (fecal samples of captive individuals fed with SW in Group N). Fresh *Oxytropis ochrocephala* that grew near the sampling sites were harvested and SW extraction was performed by ultrasonic-chloroform extraction method to a final purity of ~93% [38]. Then, it was dissolved in distilled water at a concentration of 1 mg/10 mL, sprayed evenly on the weighed feed, and dried for packing into self-sealing bags [26]. The dosage of SW was determined based on the daily intake of *Oxytropis* by plateau pika [33,34]. Accordingly, 0.1 mg SW was added to the daily feed of each individual.

### 2.4. DNA Extraction and Sequencing

DNA from the fecal material was extracted using a QIAamp DNA Stool Mini Kit (QIAGEN, Dusseldorf, Germany) following the standard protocol. DNA concentration was determined using a NanoDrop ND-1000 (Thermo Fisher Scientific, Waltham, MA, USA). The V3 and V4 regions of 16S rDNA from all samples were amplified using the 341F (5′-CCTAYGGGRBGCASCAG-3′) and 806R (5′-GGACTACNNGGGTATCTAAT-3′) primers. The polymerase chain reaction (PCR) products were quantified and purified using a QuantiFluor™ fluorometer (Promega Biotech, Madison, WI, USA). Negative controls included no templates for DNA extraction and PCR amplification. PCR products were mixed in equidensity ratios. The mixture of PCR products was purified with a Gel Extraction Kit (QIAGEN). Sequencing libraries were generated using the TruSeq^®^ DNA PCR-Free Sample Preparation Kit (Illumina, San Diego, CA, USA) following manufacturer’s instructions and index codes were added. The library quality was assessed on the Qubit@ 2.0 Fluorometer (Thermo Fisher Scientific) and the Bioanalyzer 2100 system (Agilent, Santa Clara, CA, USA). Finally, the library was sequenced on a HiSeq2500 platform (Illumina) and 250 bp paired-end reads were generated.

### 2.5. Bioinformatics and Statistical Analysis

Sequences were normalized, filtered, and processed by QIIME v 1.9.1 [39]; only sequences with an average quality score >25 were included in the analysis. The original data were pooled together and all effective reads were searched against the Greengenes 13.5 reference database [40] and clustered into operation taxonomic units (OTU) at 97% identity according to QIIME protocols. Representative OTUs were classified using Pynast, and their taxonomy (phylum, class, order, family, and genus) was assigned based on the UCLUST algorithm [41]. Thereafter, the mitochondrion and chloroplast OTUs were removed before downstream analysis. Unclassified genera were represented with an “unidentified member (U. m) of a certain taxa”, as described in a previous study [42]. Gene pathways were calculated by PICRUSt v1.1.4 [43] using the Kyoto Encyclopedia of Genes and Genomes (KEGG) database. The relative abundance of each functional hierarchy was used for analysis.

Statistical analyses were performed using R v3.5.3 and SPSS v22.0. The enterotype clustering of gut microbiota was calculated by the Partitioning around medoid (PAM) method in the R package “cluster” and the optimal number of clusters was chosen based on Calinski-Harabasz (CH) values that were calculated using the R package “clusterSim” [5]. The samples were plotted via principal coordinate analysis (PCoA) using the R package “ade4” [5]. Spearman’s correlation between genera was analyzed by the R package “psych” [44] and presented as a network using Gephi v0.9.2 [45]. SCFAs were analyzed using one-way analysis of variance (ANOVA) followed by the Dunnett’s T3 test. Relative abundance differences in genera and KEGG pathways between different enterotypes were assessed by the Kruskal–Wallis test. The similarity of percentage analysis (SIMPER) was performed by the R package “vegan” [46]. The changes of relative abundance of genera in diet treatment were processed and analyzed using STAMP v2.1.3 [47].

### 2.6. Data Availability

The 16S rDNA sequences data have been uploaded in the National Center for Biotechnology Information database using accession number of PRJNA613933.

## 3. Results

### 3.1. Enterotype Identification of Plateau Pikas

**Figure 1:**
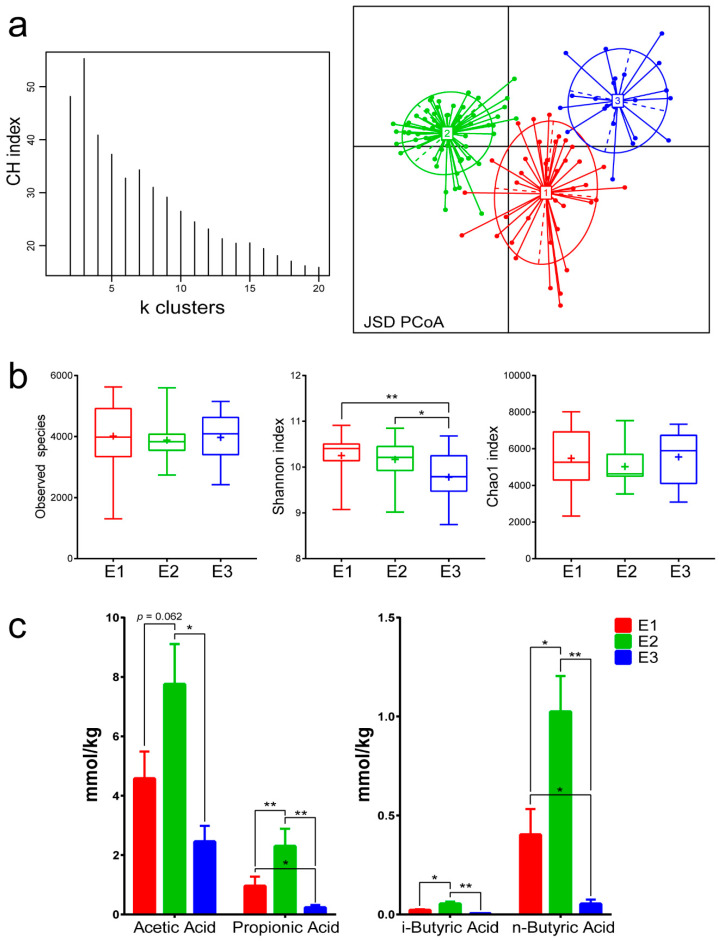
Identification of enterotypes in pika. (**a**) Calinski-Harabasz (CH) indices for a number of enterotypes and principal coordinate analysis (PCoA) analysis based on Jensen–Shannon distance (JSD) dissimilarity. (**b**) Alpha diversity (observed species, Shannon index, and Chao1 index) of the three enterotypes. Differences were assessed by the Kruskal–Wallis test and denoted as follows: * *p* < 0.05; ** *p* < 0.01. (**c**) Four major short-chain fatty acids (SCFAs) (mean ± SE) of the three enterotypes. Differences were calculated by one-way analysis of variance (ANOVA) and are denoted as follows: * *p* < 0.05; ** *p* < 0.01.

**Figure 2:**
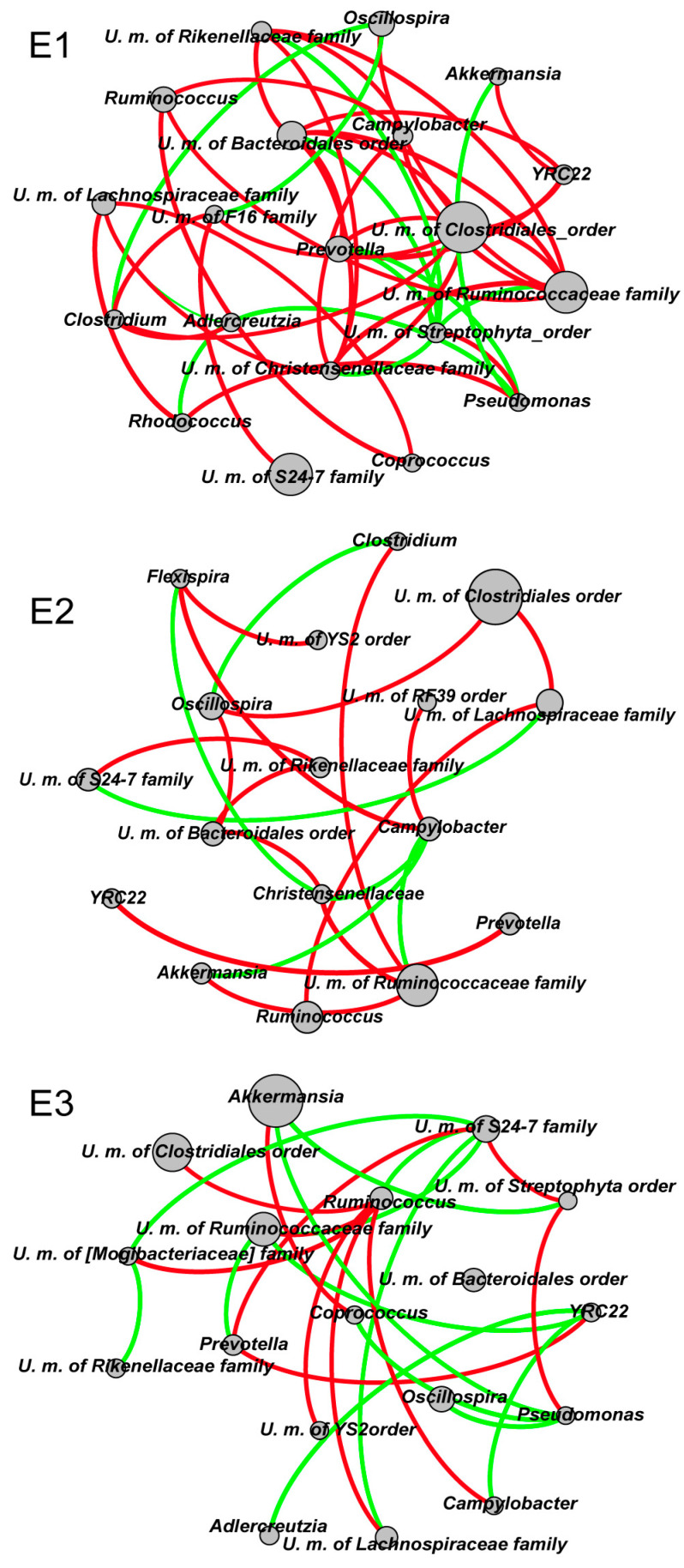
Co-occurrence networks of the top 20 genera in the three enterotypes. Spearman’s correlation greater than 0.4 or lower than −0.4 is illustrated and line color reflects direction (green: negative; red: positive).

We calculated Jensen–Shannon distances (JSDs) for genus-level relative abundance profiles, and the highest CH index value was obtained for three clusters. The analysis was repeated with the Bray–Curtis and Euclidean distance metrics and found that the optimal number of clusters was three as well (Figure S1). The JSD [5] PCoA was used to divide and display the three enterotypes (Figure 1a). In total, of the 126 samples, 40 (31.7%) were assigned to enterotype 1 (E1), 64 (50.8%) to enterotype 2 (E2), and 22 (17.5%) to enterotype 3 (E3). E1 and E2 were distributed throughout the sampling points, whereas E3 clustered mainly in July 2017 and April 2018 (Table S2). Three clusters of gut communities based on the relative abundance of bacterial genera enriched in these three enterotypes emerged (Table 1). The three enterotypes shared similar species composition and Chao1 index; however, E1 presented the highest Shannon index and E3 presented the lowest (Kruskal–Wallis test, χ^2^ = 12.669, *p* = 0.002) (Figure 1b). Significant differences regarding the content of SCFAs were observed between the three enterotypes as determined by one-way ANOVA tests of acetic acid (F_2,52_ = 3.731, *p* = 0.031), propionic acid (F_2,52_ = 4.283, *p* = 0.019), i-butyric acid (F_2,52_ = 8.297, *p* = 0.001), and n-butyric acid (F_2,52_ = 6.653, *p* = 0.003). E2 presented the highest SCFA content, followed by E1 and then E3 (Figure 1c). Co-occurrence networks based on the top 20 genera revealed distinct classifications of the three enterotypes (Figure 2 and Table S3). In E1, the U. m. of the Clostridiales order and the U. m. of the Ruminococcaceae family were most correlated with other genera, whereas the U. m. of the S24-7 family, despite its abundance, exhibited only one positive correlation with the U. m. of F16 family. A similar pattern was observed for *Akkermansia*, which exhibited a positive correlation with *YRC22* and a negative correlation with *Pseudomonas*. It should be noted that E1 exhibited the most complex network structure, whereas E2 had the least complex network structure. Specifically, in the E2 network, the U. m. of the S24-7 family and *Akkermansia* were less abundant than that in the other two enterotypes, and there were fewer links to the two dominant genera compared to the E1. The U. m. of the S24-7 family exhibited a positive correlation with the U. m. of the Rikenellaceae family and a negative correlation with the U. m. of the Lachnospiraceae family. *Akkermansia* exhibited a positive correlation with the U. m. of the Ruminococcaceae family and a negative correlation with that of *Campylobacter*. Finally, in the E3 network, *Akkermansia* was the most abundant but the U. m. of the S24-7 family had the most links.

### 3.2. Functional Differences between the Enterotypes

According to the results of functional prediction, we identified the major functional differences in the three enterotypes using the SIMPER method to identify the top five and top 10 signature functional categories between each of the two enterotypes from levels 2 and 3 (Table S4). A total of seven pathways at level 2 and 13 pathways at level 3 contributed most to functional differences (Table 2). At level 2, E1 was enriched with genes involved in amino acid metabolism, while E2 was enriched with those involved in carbohydrate metabolism as well as xenobiotic biodegradation and metabolism. E3 was enriched with genes involved in genetic information processing, replication and repair, and cell motility. At level 3, E1 was enriched with genes encoding amino acid-related enzymes, such as those involved in glycine, serine, threonine, alanine, aspartate, and glutamate metabolism, while E2 was enriched with genes involved in glycolysis/gluconeogenesis, butanoate metabolism, and fatty acid metabolism, and those encoding ATP-binding cassette transporters. Meanwhile, E3 was enriched with genes involved in DNA repair and replication, as well as those encoding recombination and repair proteins, bacterial motility proteins, and flagellar assembly and secretion systems.

### 3.3. Response of Dominant Genera to Swainsonine

**Figure 3:**
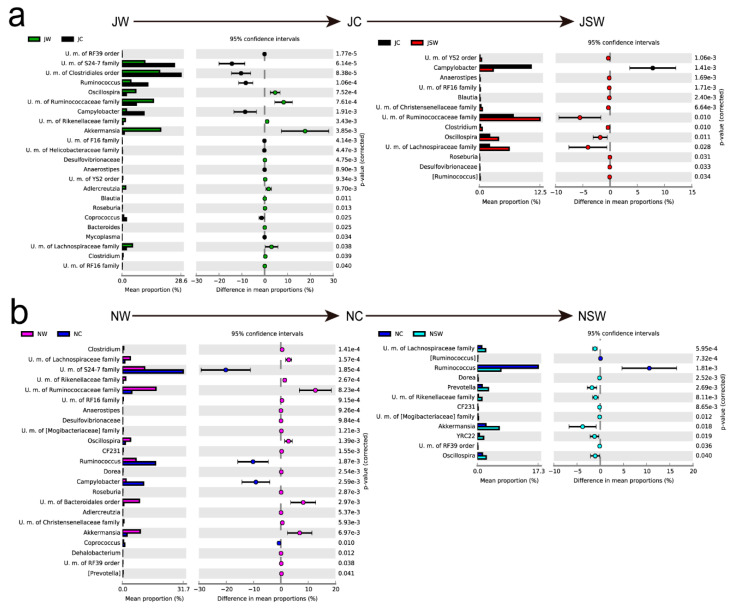
Response of dominant genera to interventions. (**a**) Changes in and resilience of genera in Group J. (**b**) Changes in and resilience of genera in Group N. Differences were assessed by the Welch’s *t*-test and denoted as the corrected *p*-value.

In Group J (Figure 3a), the relative abundance of genera such as *Akkermansia*, the U. m. of the Ruminococcaceae family, *Oscillospira*, and the U. m. of the Lachnospiraceae family were significantly reduced, while the U. m. of the S24-7 family, the U. m. of the Clostridiales order, *Ruminococcus*, and *Campylobacter* et al. were significantly increased after 20 weeks of intervention (JW-JC). However, after adding SW to their daily diet for 4 weeks, the relative abundance of the U. m. of the Ruminococcaceae family, *Oscillospira*, and the U. m. of the Lachnospiraceae family was significantly increased, while *Campylobacter* was significantly decreased (JC-JSW).

In Group N (Figure 3b), the relative abundance of genera such as *Akkermansia*, the U. m. of the Bacteroidales order, the U. m. of the Ruminococcaceae family, *Oscillospira*, and the U. m. of the Lachnospiraceae family were also significantly reduced, while the U. m. of the S24-7 family, *Ruminococcus*, and *Campylobacter* et al. were significantly increased after 2 weeks of intervention (NW-NC). Then, after adding SW to their daily diet, the relative abundance of *Akkermansia*, *Oscillospira*, and the U. m. of the Lachnospiraceae family et al. was significantly increased, while that of *Ruminococcus* was significantly decreased (NC-NSW).

The above-mentioned bacterial taxa were the major genera, as summarized in Table 1, and were found to be present in large abundance in the gut microbiota plateau pikas, thereby indicating that a diet with or without SW considerably changed the gut microbial composition.

### 3.4. Response of Enterotypes and Microbial Diversity to Swainsonine

**Figure 4:**
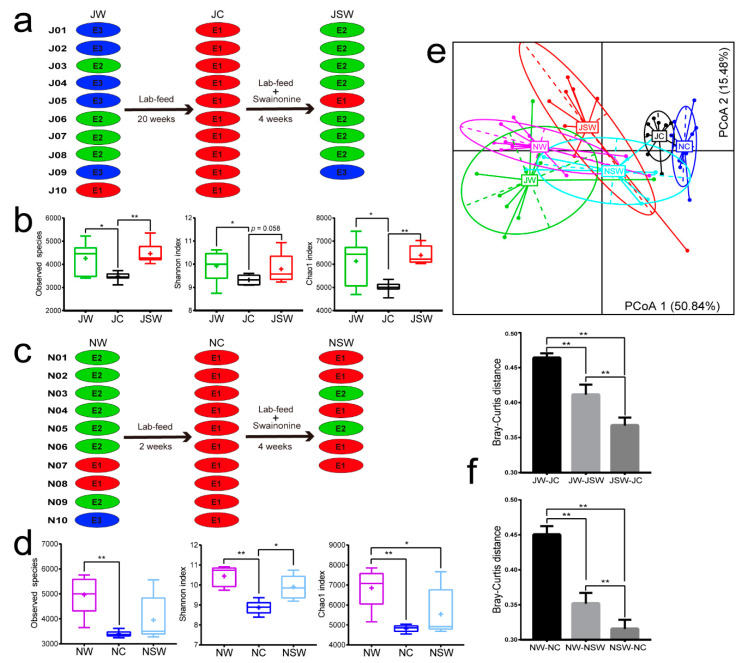
Responses of enterotypes and microbial diversity to interventions. (**a**,**b**) Changes in and resilience of enterotypes and alpha diversity after long-term dietary intervention. (**c**,**d**) Changes in and resilience of enterotypes and alpha diversity after short-term dietary intervention. (**e**) PCoA based on Bray–Curtis dissimilarity of the six groups. (**f**) Differences in Bray–Curtis dissimilarity values in Group J and Group N. Differences were calculated by the Kruskal–Wallis test and are denoted as follows: * *p* < 0.05; ** *p* < 0.01.

Enterotype clustering was performed using Group J and Group N samples. In the ten pikas captured in July 2017, enterotype clustering showed that nine individuals with initial enterotypes E2 or E3 (JW) transitioned to E1 after 20 weeks of intervention (JC). After adding SW to their daily laboratory diet (JSW), five of the nine plateau pikas reverted to the original type, while three plateau pikas whose original enterotypes were E3 changed to E2 and E1 was found in one pika (Figure 4a). In the ten plateau pikas captured in November 2017, enterotype clustering showed that eight individuals whose enterotype was initially E2 or E3 (NW) transitioned to E1 after 2 weeks of intervention (NC), indicating that this period was sufficient to change enterotypes. Of the seven plateau pikas sampled after SW addition (NSW), only two reverted to the original enterotype, while E1 was found in the other five pikas (Figure 4c).

Analysis of alpha diversity (Figure 4b,d) revealed that the diversity of gut microbiota in wild individuals decreased to a varying degree after 20 or 2 weeks of indoor rearing. Moreover, the addition of SW to the diet restored the alpha diversity of Group J to original levels (Kruskal–Wallis test, observed species: χ^2^ = 11.434, *p* = 0.003; Shannon index: χ^2^ = 6.462, *p* = 0.040; Chao1 index: χ^2^ = 11.586, *p* = 0.003), whereas the diversity of Group N achieved only partial recovery (observed species: χ^2^ = 15.771, *p* < 0.001; Shannon index: χ^2^ = 18.239, *p* < 0.001; Chao1 index: χ^2^ = 13.879, *p* = 0.001). Bray–Curtis PCoA analysis of the relative abundance of the genera from these groups revealed a clear difference between the two wild groups (JW and NW), but similarity between the intervention groups (JC and NC) was observed. This finding indicates that the gut microbiota of wild plateau pikas changed similarly after long- or short-term diet intervention during winter and summer. The characterization of Groups JSW and NSW and comparison between wild and indoor-reared individuals confirmed that SW promoted microbiota restoration to the original state (wild type) (Figure 4e). Bray–Curtis distances between the groups showed that Group JSW was closer to JC than to JW (Kruskal–Wallis test, χ^2^ = 60.706, *p* < 0.001), thereby indicating that the resilience induced by SW could not completely restore the gut microbial composition of indoor-reared pikas. Finally, similar results were observed in Group N animals as well (Kruskal–Wallis test, χ^2^ = 55.016, *p* < 0.001) (Figure 4f).

## 4. Discussion

In this study, we confirmed three enterotypes of the gut microbial community and characterized the differences in dominant genera in plateau pikas (Table 1). With regard to mammals, the dominant taxon of enterotypes is not only determined by host phylogeny but also by environmental factors. Moeller et al. found that *Acinetobacter* and unclassified Ruminococcaceae species were the dominant genera of two enterotypes in vegetarian gorillas [48]. Another study reported that these gut microbiota were dominated by environmentally derived bacterial taxa that facilitated the digestion of cellulose [49]. Ruminococcaceae are more abundant in wild deer mice (*Peromyscus maniculatus*), whereas S24-7 is more abundant in captive deer mice [50]. In our study, the dominant taxon was an unclassified genus belonging to the S24-7 family, although the genera *Prevotella* and *Clostridium* were also frequent in the E1 of plateau pikas (Table 1). Increased abundance of S24-7 has also been described in diabetes-prone mice fed with a laboratory diet [51] and its dominance has been reported during hibernation of arctic ground squirrels (*Urocitellus parryii*) [52]. In E2, the most abundant bacteria were unclassified Ruminococcaceae and an unclassified genus of the Clostridiales order. The former play an important role in cellulose digestion and energy uptake [53] and are enriched in the enterotype of gorillas as mentioned above [48]. Clostridiales is a Gram-positive order belonging to the Firmicutes phylum and includes the most important butyrate-producing microorganisms in the gut [54], such as Lachnospiraceae and *Ruminococcus* [55]. Clostridiales can produce acetate through reductive acetogenesis and butyrate or propionate through lactate utilization [56]. They are also the principal intestinal microorganisms to degrade plant carbohydrates, producing SCFAs as their main catabolites [57]. Plateau pikas with E2 also exhibited the highest SCFA value, which was associated with fatty acid production and host-absorption rates (Figure 1c). These findings suggest that plateau pikas have a stronger energy absorption than other herbivores living at lower altitudes, which helps them overcome harsher environmental conditions, including food scarcity in the plateau. The fact that similar genus-level enrichment was not found in yaks and Tibetan sheep, which share the same environment [58], could be explained by the special lifestyle of plateau pikas and their physiology that include usage of non-shivering thermogenesis to cope with extremely cold weather and maintenance of body temperature at 39–40 °C [59]. *Akkermansia*, the genus enriched in E3, is an important probiotic in the gut and has been inversely associated with obesity, diabetes, inflammation, and metabolic disorders [60]. It can also regulate mucus thickness and maintain intestinal barrier integrity [61]. E3 has been observed mainly during the green grass season rich in toxic plants, and detoxification may explain the higher content of *Akkermansia* in pikas compared to other enterotypes. The intestinal flora in pandas commonly harbor high proportions of *Pseudomonas*, which are rare in other mammals [62], and *Bacteroides* are particularly abundant in the koala gut microbiota [63]. Animals with detoxification capabilities may have evolved gut microbiomes for degrading plant toxins but the dominant bacteria are different to correspond to distinct PSCs in their natural diet.

High species diversity of gut microbiota may cause the ecosystem to become more resilient and adapt more easily to external perturbations [64]. There were significant differences in Shannon indices among the three enterotypes (Figure 1b), indicating that they were characterized by overall similarity but a distinct bacterial composition, which was in line with previous studies on the alpha diversity of gut microbiota [65]. Co-occurrence analysis revealed different correlations between genera enriched within each enterotype, thereby confirming a distinct abundance and synergistic relationship among genera. We found clear differences between the intestinal gut enterotype network in plateau pikas and humans [5,66], with the former displaying more complex network structure and more links in genera (Figure 2). It shows that there are variations in gut microbiota interactions between plateau herbivores and other species living at low altitudes, thereby indicating that environmental conditions are one of the important factors determining the microbial relationship in the gut.

There were considerable differences in microbial functions of the three enterotypes (Table 2). E1 exhibited an evident advantage with regard to amino acid metabolism, while E2 was enriched with genes involved in carbohydrate and fatty acid metabolism. These two enterotypes were more inclined toward energy utilization, confirming similar results from other plateau species and indicating that high-altitude animals have adapted in a similar way to cope with the alpine environment and food shortage [58]. E2 was second only to E1 in terms of genes belonging to the amino acid metabolism pathway but contained genes belonging to different types of metabolism, and thus E2 could produce more SCFAs than E1 (Figure 1c). It is worth noting that the laboratory feed made all the enterotypes transfer to E1 (Figure 4a,c), a higher protein content (Table S2) undoubtedly required correspondingly stronger metabolic capacity; this observation was consistent with that of other studies [17,50]. In terms of various metabolic pathways, E3 was much weaker than E1 and E2, indicating that the focus of this enterotype was not food-derived energy. On the other hand, genes enriched in E3 were mainly associated with the health of the gut flora (e.g., replication and repair in level 2, bacterial motility proteins in level 3). These functional pathways of the gut microbiota are usually related to detoxification [67,68], which confirms our conjecture about E3.

Kohl et al. (2010) have reported that, in small mammals that are reared indoors, the alpha diversity of the intestinal flora increases after adding PSCs to their artificial diet [26]. However, their study did not involve the enterotype framework. Recently, Costea et al. suggested that enterotypes were changeable and influenced by several factors, such as geography, age, and diet [14]. After long- and short-term rearing along with administration of an artificial protein-rich diet under uniform laboratory conditions, the enterotypes of plateau pikas, captured both in July and November, converged to E1 from E2 and E3 (Figure 4). This observation is consistent with the results of experiments performed on mice and deer mice [19,50]. Thus, our findings indicate that the intervention of artificial diets can cause significant compositional changes, leading to a short-term enterotype shift. However, in our study, the enterotype composition could not be restored to the original state after long-term alterations in diet without other stimulations or environmental changes. Several studies have suggested enterotype resilience but few have investigated how it may affect overall community resemblance or enterotype distribution [13,14,23]. Our results show that SW promoted enterotype resilience but did not restore enterotype compositions to the original state in all the pikas or change every individuals to E3 (Figure 4a,c), which was supposed to exhibit the advantage of detoxification. This finding was not entirely consistent with the predictions of enterotype resilience. There may be various explanations for this phenomenon. First, bacteria exhibit different growth rates. Vieira-Silva et al. reported that the Firmicutes-dominant enterotype, which is estimated to have the lowest overall bacterial growth rate [69], also exhibited a delayed return to equilibrium [14]. Similar phenomena were observed in our experiments (Figure 3), where the degree of recovery after long- and short-term interventions varied among some dominant bacteria. Second, it is likely that individual differences also play a major role in the process analogous to the unpredictable ways different individuals respond to stressors or even to the same environmental stimuli [70]. Third, the plasticity of an individual’s gut microbiota may be so limited that it would not allow for change, whereas, for others, it may morph more readily into different enterotypes. Additionally, the amount of SW added in this experiment was the usual daily intake of plateau pika, and higher levels or more kinds of PSCs may be required for further transferring the enterotypes to E3. Future PSC-based intervention studies with gradient concentration and different time periods are warranted to examine whether and how these species can finally return to the initial wild-type state.

## 5. Conclusions

In summary, we confirmed significant inter-individual variations in the gut microbiota composition and provided evidence for the existence of three compositionally different enterotype clusters in the wild population of plateau pikas. The three enterotypes had different microbial functions and the dominant genera of each cluster exhibited several characteristics associated with adapting to high altitudes. Microbial diversity was significantly reduced upon transferring plateau pikas from the field to a laboratory on a short- or long-term basis, and the three enterotypes converged to a single one with a change in the compositions of dominant genera. SW could increase the diversity of gut microbiota, bringing the microbiota structure of indoor individuals close to that of wild individuals, and thus might be critical in contributing to the restoration of natural enterotypes. Our findings lay a foundation for future in-depth research to better understand the impact of PSC–microbiome interactions on the lifestyle of host mammals in various environmental conditions.

## Figures and Tables

**Table 1:** Average relative abundances of the major genera (top 15) among the three enterotypes. *p*-values had been corrected using the false discovery rate control. All pairwise analysis of the Kruskal–Wallis test was used for post hoc multiple comparisons and significant differences were denoted as: E1 vs. E2, * *p* < 0.05, ** *p* < 0.01; E1 vs. E3, # *p* < 0.05, ## *p* < 0.01; E2 vs. E3, ▲ *p* < 0.05, ▲▲ *p* < 0.01.

Major Genera	Relative Abundance			Kruskal–Wallis	
	E1	E2	E3	χ^2^	Corrected *p*-Value
U. m. of Clostridiales order	0.149609	0.231802 **^▲▲^	0.134758	71.450	<0.01
U. m. of Ruminococcaceae family	0.089191	0.148726 **^▲▲^	0.106976	44.657	<0.01
U. m. of S24-7 family	0.106665 **^##^	0.040452	0.059563 ^▲▲^	65.332	<0.01
*Akkermansia*	0.045254	0.017659 **	0.226423 ^##▲▲^	68.805	<0.01
*Ruminococcus*	0.035577	0.078697 **^▲▲^	0.032629	68.675	<0.01
*Oscillospira*	0.037339	0.054031 **	0.052206 ^#^	17.490	<0.01
U. m. of Lachnospiraceae family	0.020661	0.053863 **^▲▲^	0.028579	61.675	<0.01
U. m. of Bacteroidales order	0.039277	0.036844	0.038935	0.075	0.963
*Prevotella*	0.039454 ^#^	0.025026	0.021277	7.640	0.022
*Campylobacter*	0.013517 ^#^	0.035719 **^▲▲^	0.003411	38.725	<0.01
U. m. of Rikenellaceae family	0.012253	0.013115 ^▲^	0.009776	6.518	0.038
*YRC22*	0.011418	0.007679	0.008951	2.834	0.242
*Coprococcus*	0.005219	0.010096 **^▲▲^	0.005537	37.595	<0.01
U. m. of Streptophyta order	0.013972 **^##^	0.003364	0.003211	14.531	<0.01
*Clostridium*	0.008206 *^##^	0.005554	0.005075	9.839	<0.01

**Table 2:** Average relative abundances of hierarchical functional categories (Kyoto Encyclopedia of Genes and Genomes (KEGG) pathway levels 2 and 3) among the three enterotypes. *p*-values had been corrected using the false discovery rate control. All pairwise analysis of the Kruskal–Wallis test was used for post hoc multiple comparisons and significant differences were denoted as: E1 vs. E2, * *p* < 0.05, ** *p* < 0.01; E1 vs. E3, # *p* < 0.05, ## *p* < 0.01; E2 vs. E3, ▲ *p* < 0.05, ▲▲ *p* < 0.01.

Level	Pathways	Relative Abundance			Kruskal–Wallis	
		E1	E2	E3	χ^2^	Corrected *p*-Value
	Carbohydrate metabolism	0.0965	0.1060 **^▲▲^	0.0954	90.779	<0.01
Level 2	Amino acid metabolism	0.1087 **^##^	0.1000 ^▲^	0.0964	86.083	<0.01
	Genetic information processing	0.0255 **	0.0241	0.0300 ^##▲▲^	67.444	<0.01
	Replication and repair	0.0729	0.0740	0.0803 ^##▲▲^	49.192	<0.01
	Cell motility	0.0328 **	0.0292	0.0358 ^▲▲^	27.391	<0.01
	Xenobiotics biodegradation and metabolism	0.0281	0.0299 ^▲▲^	0.0262	12.437	<0.01
	Membrane transport	0.1017	0.1027	0.1001	5.254	0.072
	Amino acid-related enzymes	0.0171 **^##^	0.0134 ^▲▲^	0.0119	97.151	<0.01
Level 3	Glycolysis/Gluconeogenesis	0.0103	0.0146 **^▲▲^	0.0101	95.062	<0.01
	Butanoate metabolism	0.0075	0.0107 **^▲▲^	0.0077	90.177	<0.01
	Glycine, serine, and threonine metabolism	0.0099 **^##^	0.0081	0.0079	82.622	<0.01
	Alanine, aspartate, and glutamate metabolism	0.0109 **^##^	0.0084	0.0083	81.392	<0.01
	Fatty acid metabolism	0.0055	0.0077 **^▲▲^	0.0056	76.405	<0.01
	DNA repair and recombination proteins	0.0228	0.0238 *	0.0282 ^##▲▲^	69.998	<0.01
	Replication, recombination, and repair proteins	0.0068	0.0065	0.0101 ^##▲▲^	57.479	<0.01
	Bacterial motility proteins	0.0172 *	0.0153	0.0191 ^##▲▲^	30.042	<0.01
	Flagellar assembly	0.0071 *	0.0060	0.0079 ^▲▲^	20.758	<0.01
	Secretion system	0.0177	0.0173	0.0190 ^##▲▲^	15.086	<0.01
	Transporters	0.0467 ^#^	0.0479 ^▲▲^	0.0447	11.341	<0.01
	ATP-binding cassette transporters	0.0286	0.0291 ^▲▲^	0.0272	11.122	<0.01

## Supplementary Materials

The following are available online at https://www.mdpi.com/2076-2607/8/9/1311/s1, Figure S1: CH index based on the Bray–Curtis and Jaccard distance metrics, Table S1: Main components of the pika feed, Table S2: Distribution of enterotypes with respect to sampling time, Table S3: Network indices of the three enterotypes in plateau pikas, Table S4: Contribution of hierarchical functional categories (KEGG pathway levels 2 and 3) to total variance among the three enterotypes.
