# Supplementary material for: Enterotypes of the Gut Microbial Community and Their Response to Plant Secondary Compounds in Plateau Pikas
Source: Microorganisms. 2020 Aug 28;8(9):1311. doi: 10.3390/microorganisms8091311 (PMC7563992; doi:10.3390/microorganisms8091311)
Supplement: Supplementary file 1 [file microorganisms-08-01311-s001.pdf]

Figure S1. CH index based on the Bray-Curtis and Jaccard distance metrics.

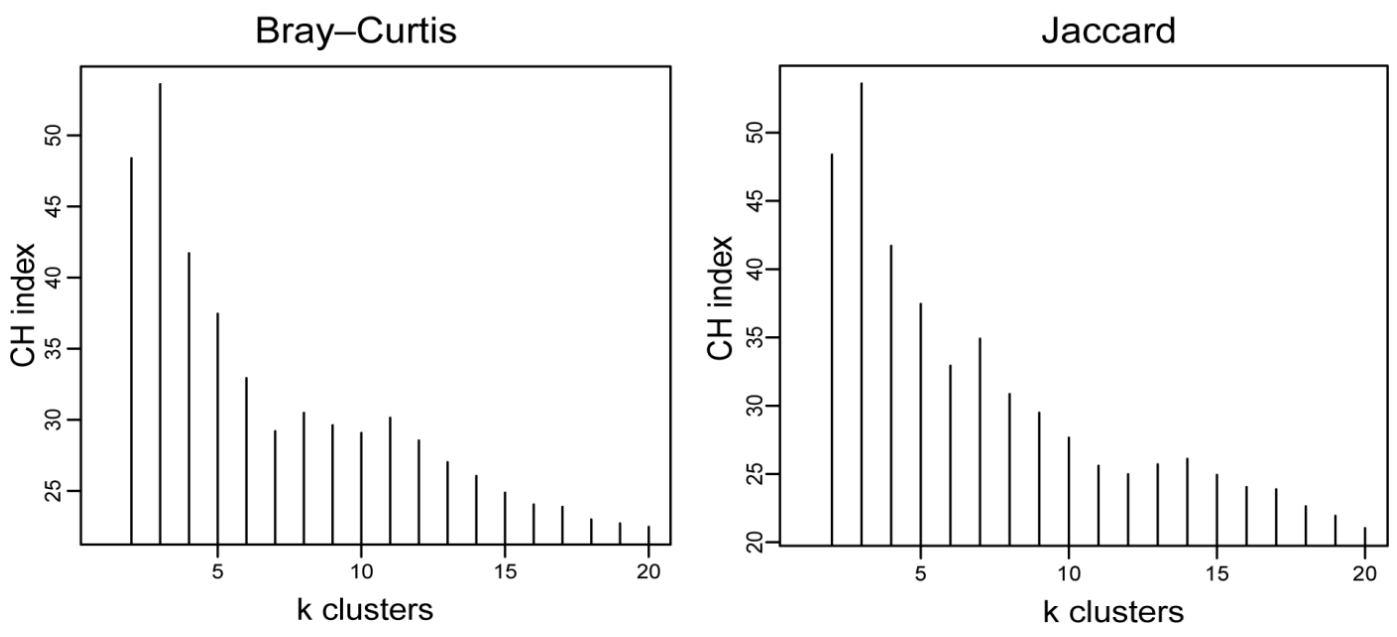

Table S1. Main components of the pika feed.

	Main food material	Main nutrition contents
Laboratory	*Zea mays*, Soybean meal, Lucerne meal, Salt, Dicalcium phosphate, Mountain flour, et al.	Crude protein: 16.6%<br>Crude fat: 3.1%<br>Crude fiber: 11.2%<br>Crude ash: 8.5%<br>Nitrogen-free extract: 52.8%
Natural	**July**: *Elymus nutans*, *Festuca ovina*, *Poa annua*, *Kobresia humilis*, *Potentilla anserina*, *Oxytropis ochrocephala*, et al.<br>**November**: *Elymus nutans*, *Oxytropis ochrocephala*, *Kobresia humilis*, *Scirpus distigmaticus*, *Poa annua*, *Leontopodium pusillum*, et al.	**July**: Crude protein: 13.3%<br>Crude fat: 2.3%<br>Crude fiber: 25.0%<br>Lignin: 19.3%<br>**November**: Crude protein: 8.0%<br>Crude fat: 4.2%<br>Crude fiber: 22.0%<br>Lignin: 19.9%

Table S2. Distribution of enterotypes with respect to sampling time.

Sampling time	E1	E2	E3
July 2015	1	8	0
July 2016	8	4	1
January 2017	4	8	0
April 2017	1	12	0
July 2017	3	7	16
November 2017	9	3	0
January 2018	1	19	0
April 2018	7	1	5
July 2018	6	2	0

Table S3. Network indices of three enterotypes in plateau pikas.

Network indices	E1	E2	E3
No. of samples	40	64	22
Total nodes	20	17	18
Total links	45	20	24
Positive links	32	14	11
Negative links	13	6	13
Links per node	2.250	1.176	1.333
Average Degree	4.500	2.353	2.667
Average Weighted Degree	2.186	0.834	-0.038
Diameter	5	7	6
Average path length	2.216	2.925	2.779
Density	0.237	0.147	0.157
Average Clustering Coefficient	0.411	0.192	0.451
Total triangles	31	3	7

Table S4. Contribution of hierarchical functional categories (KEGG pathway levels 2 and 3) to total variance among the three enterotypes. Values were calculated via the SIMPER method.

Level	E1 vs E2		E1 vs E3		E2 vs E3	
	Pathways	Contribution to variance	Pathways	Contribution to variance	Pathways	Contribution to variance
Level 2	Carbohydrate metabolism	0.127	Amino acid metabolism	0.164	Carbohydrate metabolism	0.130
	Amino acid metabolism	0.118	Replication and repair	0.107	Cell motility	0.098
	Membrane transport	0.093	Membrane transport	0.092	Membrane transport	0.081
	Cell motility	0.081	Cell motility	0.078	Replication and repair	0.079
	Xenobiotics biodegradation and metabolism	0.060	Genetic information processing	0.060	Genetic information processing	0.072
Level 3	Transporters	0.041	DNA repair and recombination proteins	0.048	Transporters	0.043
	Glycolysis / Gluconeogenesis	0.037	Transporters	0.048	Glycolysis / Gluconeogenesis	0.038
	Amino acid related enzymes	0.032	Amino acid related enzymes	0.047	DNA repair and recombination proteins	0.037
	Butanoate metabolism	0.028	ABC transporters	0.031	Bacterial motility proteins	0.037
	ABC transporters	0.027	Replication, recombination and repair proteins	0.029	Replication, recombination and repair proteins	0.030
	Bacterial motility proteins	0.026	Bacterial motility proteins	0.029	ABC transporters	0.027
	Alanine, aspartate and glutamate metabolism	0.022	Alanine, aspartate and glutamate metabolism	0.024	Butanoate metabolism	0.025
	General function prediction only	0.021	Secretion system	0.020	Flagellar assembly	0.021
	Fatty acid metabolism	0.019	Glycine, serine and threonine metabolism	0.018	Secretion system	0.020
	Secretion system	0.017	Flagellar assembly	0.017	Fatty acid metabolism	0.018
